# Determinants of undernutrition among settled pastoralists' children aged 6–59 months in Kenya

**DOI:** 10.1002/fsn3.4201

**Published:** 2024-05-19

**Authors:** Amos Otieno Adongo, Joseph Wafula Matofari, Elizabeth Kamau Mbuthia

**Affiliations:** ^1^ Department of Dairy, Food Science and Technology (DAFTEC) Egerton University Njoro Campus Egerton Kenya; ^2^ Kenya Agricultural and Livestock Research Organization (KALRO) Sheep, Goat & Camel Research Institute Marsabit Kenya; ^3^ Department of Human Nutrition Egerton University Njoro Campus Egerton Kenya

**Keywords:** children, pastoralist, stunting, underweight, wasting

## Abstract

The transition from nomadism to sedentary lifestyle has introduced changes in diets and undernutrition is endemic among settled pastoral households. This study aimed to investigate the underlying factors affecting stunting, underweight, and wasting of children aged 6–59 months in Marsabit County, Kenya. A cross‐sectional household survey was conducted in six wards capturing pastoral, agro‐pastoral, and urban livelihood practices. Using multistage sampling method, 394 children aged 6–59 months participated with written consent from the caregivers. A pretested questionnaire and anthropometric measures were used during data collection. Population characteristics were summarized into means and proportions, while chi‐square and analysis of variance were used to evaluate associations between variables. Backward logistic regressions were used to explore predictors of stunting, underweight, and wasting, respectively. The results showed that the mean Height for Age *Z*‐score, Weight for Age *Z*‐score, and Weight for Height *Z*‐score were −1.51, 1.54, and 1.02, respectively. The prevalence of stunting, underweight, and wasting was 38.1%, 23.0%, and 18.5%, respectively. The age of child, source of drinking water, and waste disposal were some of the main factors influencing stunting among children. In conclusion, the prevalence of undernutrition was high compared to the World Health Organization recommended cutoffs. Water sources hygiene, and caregiver's income were some of the main predictors of undernutrition among children. Development agencies need to focus on the supply of potable water, access to toilet facilities, in addition to nutrition education on hygienic complementary feeding practices among pastoral caregivers.

## Graphical abstract

As nomadic households settle in urban centers and satellite camps under poor sanitary conditions, challenges such as malnutrition abound. The main factors undermining a child's nutritional status included the age of child, source of drinking water, fecal waste disposal, and caregiver's income. There is need to improve the phytosanitary conditions, such as increased access to toilets and supply of potable water for domestic use in areas occupied by these nomadic households.

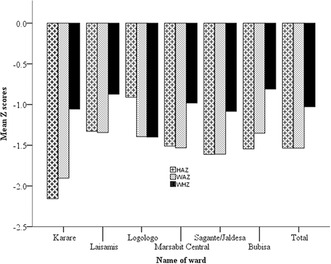

## 1 INTRODUCTION

The traditional diet of pastoralists has always included animal‐based foods, including milk, meat, and occasionally blood supplemented with wild fruits and vegetables in wet seasons (Oniang'o et al., 2003). However, recurring droughts that cause livestock death have prolonged high poverty levels, forcing nomadic people to adopt sedentary lifestyles (Asiimwe et al., 2020; Burns et al., 2022; KNBS & ICF, 2023). With occasional assistance from famine relief food, the rising sedentary pastoralists have changed their diets away from animal‐based foods such as milk and blood toward purchased goods, particularly grains and pulses. While all age groups within a household consume the same food, complementary meal preparation and handling for infants and toddlers between the ages of 6 and 59 months differ from those for adults in terms of the degree of processing and even feeding practices. One of the main causes of undernutrition, which manifests as stunting, wasting, and underweight in children aged 6–59 months, pertains to suboptimal supplemental feeding practices by caregivers.

Globally, 22% (or 148.1 million) of children under 5 years are stunted, and 6.8% (or 45.0 million) are wasted due to malnutrition (WHO, UNICEF, World Bank, 2021). This is an increase in wasting from 6.7% (45.4 million; WHO, UNICEF, World Bank, 2021). Malnutrition in sub‐Saharan Africa, Southeast Asia, and the Eastern Mediterranean continues to have high rates, accounting for 50% of child fatalities (de Onis & Branca, 2016; UNICEF, 2019). The prevalence of stunting is 30.6% in sub‐Saharan Africa compared to 30.5 and 26% in East Asia and Southeast Asia, respectively (WHO, UNICEF, World Bank, 2023). Although the prevalence of stunting and underweight in Kenya decreased from 26% and 11%, respectively (KNBS, MOH/Kenya, 2015), to 18% and 10%, respectively (KNBS & ICF, 2023), pockets of pastoral Arid and Semi‐Arid Land still have a high prevalence of stunting in areas, such as West Pokot (34%), Turkana (23%), and Marsabit (19%) (KNBS & ICF, 2023).

The impact of child undernutrition on under‐five children's linear growth, health, and cognitive development remains a public health problem (Kassa et al., 2016; Matonti et al., 2020). However, current research has limited information on the effect of settlement on underlying causes of undernutrition in this population. Several studies have found a link between children's nutritional status and underlying variables. Studies conducted in Uganda found that being a male and falling into certain age brackets, as well as poor sanitation, increased the odds of wasting among children under the age of 5 (Kinyoki et al., 2015; Obeng‐Amoako et al., 2021; Okidi et al., 2022). Other factors linked to child malnutrition include parental income and education levels (Khan et al., 2019). While data on children's nutritional status have been collected for emergency treatments in pastoral areas, there is a scarcity of knowledge on the underlying factors influencing the nutritional status of children, particularly among settled pastoralists. Therefore, the purpose of this study was to assess the prevalence of nutritional status and its associated underlying determinants in settled pastoral children in northern Kenya using Marsabit County as a case study. The findings of this study can inform policy formulation aimed at strengthening nutrition‐sensitive development initiatives among settling pastoral communities.

### 1.1 Key messages

- As nomadic households settle in urban centers and satellite camps under poor sanitary conditions, challenges such as malnutrition abound.
- The main factors undermining child nutritional status included the age of child, source of drinking water, fecal waste disposal, and caregiver's income.
- There is need to improve the phytosanitary conditions such as increased access to toilets and supply of potable water for domestic use in areas occupied by these nomadic households.

## 2 MATERIALS AND METHODS

### 2.1 Design and study population

**FIGURE 1:**
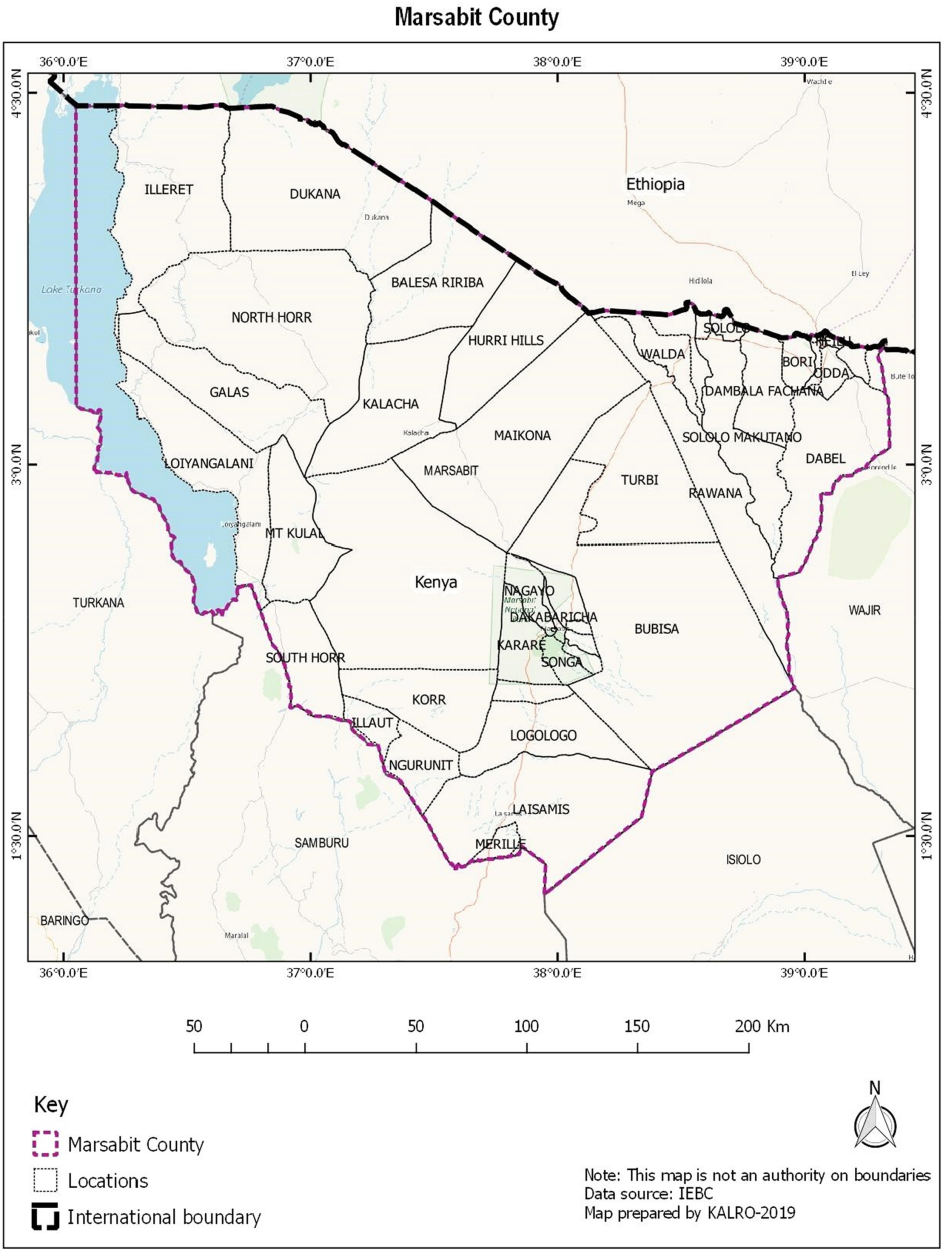
Map of Marsabit County showing study sites.

A cross‐sectional study of settled pastoral households with children aged 6–59 months was performed. The research was carried out in Marsabit County, northern Kenya (Figure 1), in the months of October and November, 2020. The research was carried out in the wards of Laisamis, Logologo, Karare, Central, Sagante/Jaldesa, and Bubisa, which reflected distinct agro‐ecological zones, social, cultural, and livelihood patterns. Children under the age of 6 months, as well as those who were chronically or terminally ill, were excluded from participating in the study.

### 2.2 Sampling procedure

The sample size was determined following the methodology proposed by Magnani (1997), focusing on a critical indicator, in this case, a minimum meal frequency of 49% in Moyale subcounty (Opiyo, 2018) with a 10% expected change in minimum meal frequency. The study used a multistage sampling method outlined by Mugenda and Mugenda (1999). A total of 394 households with an index child aged 6–59 months were selected. The first stage involved purposive selection of counties, subcounties (Laisamis, Saku, and North horr), and wards (Karare, Laisamis, Logologo, Marsabit Central, Sagante/Jaldesa, and Bubisa). Simple random sampling was then used to choose sampling clusters at the location level. A list of target children from various locations within each of the six wards was then compiled with the support of health officials and local leaders conversant with the study areas. The number of index children per ward was allocated proportionately to the population size and formed the sampling frame. In cases where more than one child between 2 and 5 years of age was encountered, the index child selected was the child aged 6 months and be the last born among the under‐five. Additionally, in cases of twins, their names were written in separate papers, folded, and the caregiver given a chance to pick one paper with a name. A systematic sampling procedure was employed, in which each household served as a sampling unit. The consent form was translated into Kiswahili and orally translated into local languages when necessary.

### 2.3 Social, economic, and demographic questionnaire

Data pertaining to children, including their birth date, birth weight, sex, and vaccination status, were gathered from their health clinic records. In addition, a pretested semi‐structured questionnaire was administered to the child's caregiver to obtain information regarding parental education levels, occupation, marital status, family size, and water and sanitation. The questionnaire also covered topics such as livestock ownership and income source.

### 2.4 Anthropometric measurements

Anthropometric measurements were conducted in accordance with the guidelines provided by the World Health Organization (WHO, 2006). The children's recumbent length or height was measured using a portable stadiometer (UNICEF, United Nations Children's fund), ensuring accuracy to the nearest 0.1 cm, as recommended by UNICEF. Weight measurements were obtained using a standard electronic scale (seca GmbH & Co. KG, Hamburg, Germany), with precision up to the nearest 100 g.

### 2.5 Data analysis

The data cleaning, management, and analysis were conducted using SPSS software program version 22 (IBM Statistics in Chicago, IL, USA). Age, weight, and height measurements were classified into *Z*‐scores (WHO, 2006), which included weight‐for‐age <‐2SD (underweight), height‐for‐age <‐2SD (stunting), and weight‐for‐height <‐2SD (wasting). The level of undernutrition was further categorized as normal (≥‐2SD), moderate (<−2 to ≥3 SD), and severe (<‐3SD). Descriptive statistics, such as means, frequencies, and standard errors of mean (SEM), were used to summarize population characteristics. Population differences were assessed using chi‐square tests and analysis of variance (ANOVA). Tukey's honest significant difference tests were employed to determine mean separations. Backward logistic regression analysis was performed to investigate the potential factors influencing stunting, underweight, and wasting as outcome variables and presented as crude and adjusted odds ratios. The outcome variables were examined for the general population and separately for each ward. All data were analyzed at the 95% confidence level.

## 3 RESULTS

### 3.1 Population characteristics

Table 1 provides a summary of the population characteristics in the six selected administrative wards of Marsabit County. A total of 394 caregivers with children aged 6–59 months were interviewed, with the highest representation in the Sagante/Jaldesa ward (29.9%) and the lowest in the Logologo ward (10.9%). The majority of households were headed by males (86.8%). Female‐headed households were high in the Marsabit Central and Sagante/Jaldesa wards compared to the lowland wards of Bubisa, Logologo, Laisamis, and Karare. The mean family size was 5.2, with significant differences across the wards (*p* < .001). The majority of caregivers (87%) were married women, with a mean age of 28.9 years (*p* > .05). The illiteracy levels were higher among caregivers (66%) than among household heads (53.3%). The illiteracy level was significantly high in the Logologo, Karare, and Bubisa wards (*p* < .001).

**TABLE 1:** Mean ± SEM of population characteristics in different wards in Marsabit County.

Household characteristics	Name of wards						Total (*N* = 394)	*p* value
	Karare (*n* = 53)	Laisamis (*n* = 69)	Logologo (*n* = 43)	Central (*n* = 62)	Sagante/Jaldesa (*n* = 118)	Bubisa (*n* = 49)		
Household head's age (years)	37.13 ± 11.0	34.94 ± 8.5	38.33 ± 11.0	36.35 ± 8.66	36.48 ± 7.62	39.02 ± 9.4	36.79 ± 9.1	.194
Caregiver's age (years)	29.79 ± 0.98	27.15 ± 0.73	29.28 ± 0.94	28.77 ± 0.85	29.22 ± 0.56	29.55 ± 1.23	28.91 ± 0.34	.256
Family size	5.8 ± 0.2^b^	4.4 ± 0.2^a^	5.6 ± 0.2^b^	5.4 ± 0.3^b^	5.2 ± 0.2^ab^	5.0 ± 0.2^ab^	5.2 ± 0.1	<.001
Weight (kg)	10.39 ± 0.22	10.07 ± 0.21	9.35 ± 0.25	9.82 ± 0.28	10.10 ± 0.18	9.60 ± 0.28	9.95 ± 0.10	.07
Height (cm)	83.74 ± 1.01^b^	81.5 ± 0.84^ab^	79.90 ± 1.04^ab^	80.52 ± 1.05^ab^	82.32 ± 0.77^ab^	78.83 ± 1.16^a^	81.39 ± 0.40	.017
Child's age (months)	30.43 ± 1.55^b^	24.66 ± 1.4^ab^	21.03 ± 1.48^a^	24.21 ± 1.6^a^	26.91 ± 1.23^ab^	21.41 ± 1.61^a^	25.24 ± 0.62	<.001
Time to water source (min)	116.79 ± 10.26^d^	41.30 ± 3.59^ab^	28.02 ± 2.31^a^	62.52 ± 6.80^bc^	46.38 ± 3.2^ab^	69.49 ± 8.46^bc^	58.37 ± 2.68	<.001
Daily water use	58.49 ± 4.48^ab^	54.78 ± 2.10^a^	79.07 ± 6.28^c^	70.32 ± 3.75b^c^	60.00 ± 2.29^ab^	48.57 ± 3.60^a^	61.17 ± 1.47	<.001

*Note*: Values with the same letters are not significantly different at *p* < .05.

Livestock rearing was the primary occupation in the majority of households (38.7%), with a high percentage observed in the lowland wards of Logologo (69%), Bubisa (65.3%), Karare (58%), Laisamis (56.5%), and Sagante/Jaldesa (17.8%), while there were no households engaged in livestock rearing in the urban Central ward. Alternative sources of livelihood included labor and self‐employment in small and medium business ventures (16.8%). Approximately 11.7% of household heads were formally employed in government entities and local nongovernmental organizations (NGOs). Formal employment was more prevalent in Marsabit Central, located in the county headquarters, and the peri‐urban wards of Sagante/Jaldesa. The unemployment rate was 10.7% and was significantly higher in Sagante/Jaldesa, followed by the Marsabit Central, Karare, Laisamis, Bubisa, and Logologo wards (*p* < .001).

The majority of caregivers (67.8%) fell within the lowest monthly income bracket of less than KES 5000.00 (USD 33.0; *p* < .001). Sagante/Jaldesa, Central, and Karare accounted for the highest number of caregivers within this income bracket, followed by Logologo, Bubisa, and Laisamis, respectively. The majority (99.5%) of caregivers practiced breastfeeding. Marsabit Central, Sagante/Jaldesa, and Logologo reported relatively less compliance in breastfeeding. Approximately 91.4% of caregivers washed their hands before feeding a child. The highest compliance with hand washing before feeding was observed in Bubisa (100%), followed by Central, Sagante/Jaldesa, Logologo, Karare, and Laisamis (Table 1). The mean weight of the index child was 7.40 ± 0.08 kg (6–11 months), 9.12 ± 0.08 kg (12–23 months), 10.71 ± .0.09 kg (24–35 months), 11.83 ± 0.12 kg (35–47 months), and 13.65 ± 0.28 kg at age group 48–59 months, with significant (*p* < .001) differences being observed (Table 1).

In terms of age categories, 49.0% of the children fell within the age bracket of 6–23 months, 30.2% in the age bracket of 24–35 months, 16% in the bracket of 36–47 months, and 4.8% in the bracket of 48–59 months. Significant differences (*p* < .001) in diarrheal prevalence were also observed among various wards. Bubisa exhibited the highest prevalence at 69.4%, followed by Laisamis, Karare, and Logologo, respectively (Table 1).

### 3.2 Water and sanitation

**FIGURE 2:**
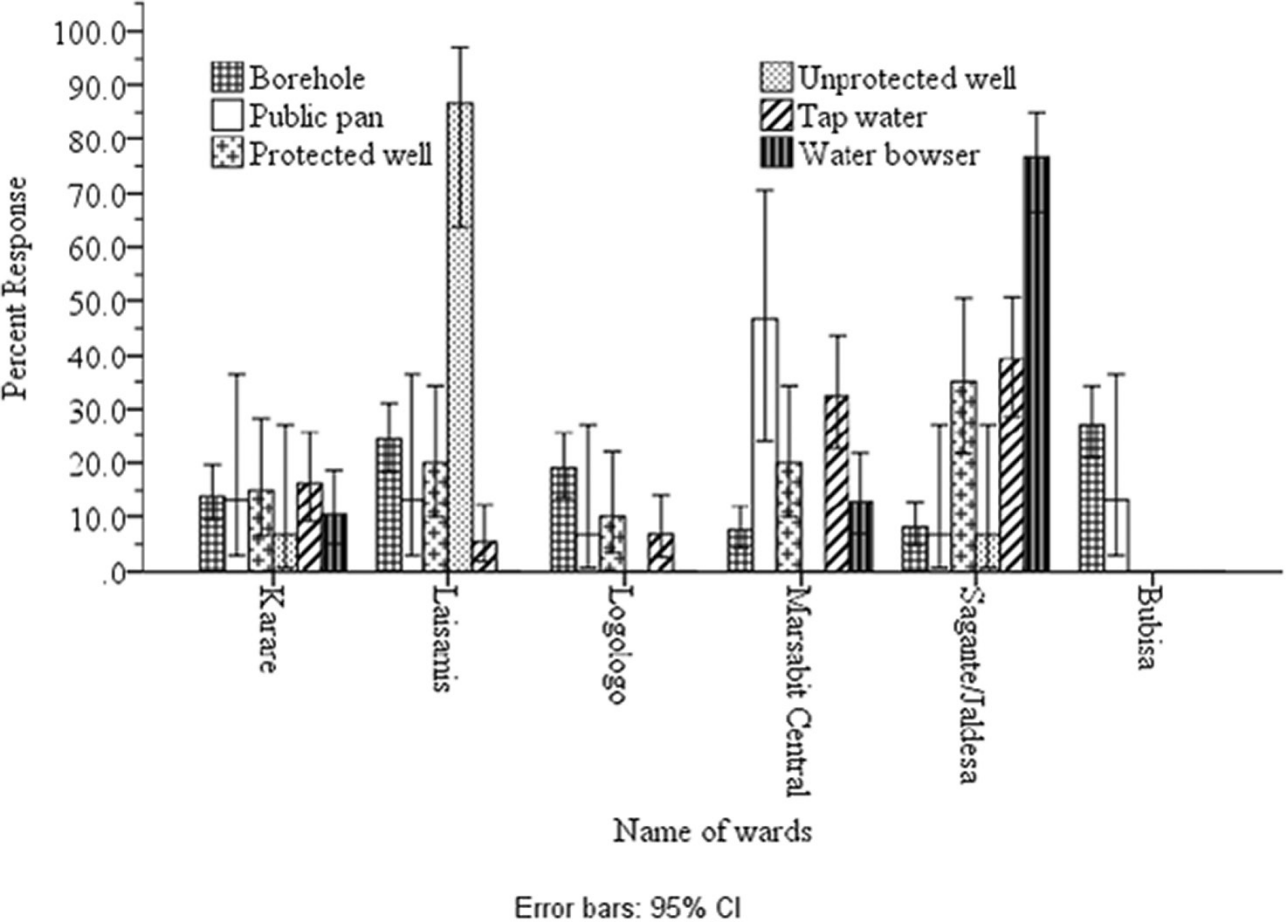
Main drinking water sources wards (*N* = 394). Error bars represent the 95% confidence interval: *χ* ^2^; < .001.

**FIGURE 3:**
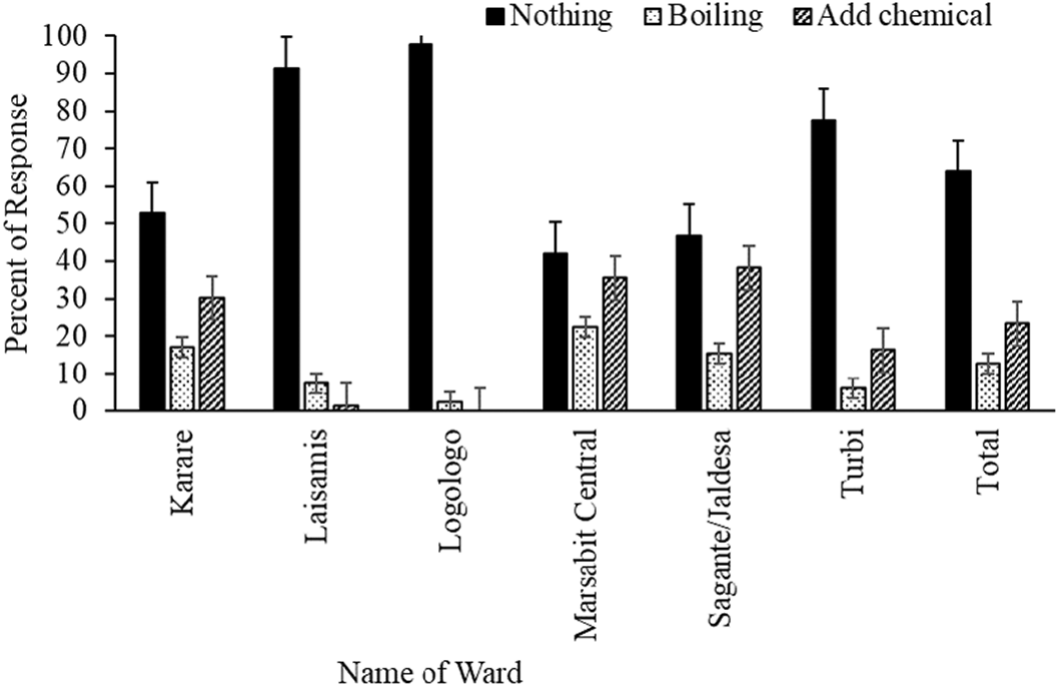
Percentage of households using different treatment methods for drinking water by ward (*N* = 394: *χ* ^2^; *p* < .001).

Figure 3 provides a summary of the main sources of water for drinking. Borehole water is the most common source, used by 43.9% of households, followed by tracked (*bowsers*) water (19.5%), tap water (18.8%), protected wells (10.2%), public water pans (3.8%), and unprotected wells (3.8%). The utilization of borehole water for drinking was significantly (*p* < .001) higher in the Bubisa (95.9%) than in the Logologo (76.7%), Laisamis (60.9%), Karare (45.3%), Central (21%), and Sagante/Jaldesa (11%) wards. Borehole water was tracked to other areas, such as in Marsabit Central (50%) and Sagante/Jaldesa (16.1%), with limited usage in the Karare ward. Borehole water was also supplied through pipes in Marsabit Central (38.7%), Sagante/Jaldesa (24.6%), Karare (22.6%), Logologo (11.6%), and Laisamis (5.8%). The use of unprotected wells as a source of drinking water was primarily observed in Laisamis (18.8%), Karare, and Sagante/Jaldesa. Protected wells were also used for drinking water in Marsabit Central (12.9%) compared to Sagante/Jaldesa (11.9%), Laisamis (11.6%), Karare (11.3%), Logologo (9.3%), and no usage in the Bubisa ward. Public water pans were common in Sagante/Jaldesa (Figure 2). The predominance of boreholes as the primary source of water highlights the importance of groundwater access in these regions and risks of exposure to human‐related water contaminants postharvesting.

Three methods of water treatment were observed: boiling (12.7%), use of chemicals (23.4%), and decanting (0.3%). Across the wards, 63.7% of households consumed untreated water (Figure 3). The consumption of untreated water was significantly higher in the Logologo (97.7%) ward, followed by Laisamis (91.3%) and Bubisa (77.6%). Furthermore, 52.8%, 46.6%, and 41.6% of households in Karare, Sagante/Jaldesa, and Marsabit Central, respectively, did not treat water before drinking (Figure 3).

Table 1 provides a summary of household access to and utilization of water in the study areas. The daily average time taken to collect water was 58.37 minutes (*p* < .001). Caregivers in the Karare ward spent significantly more time accessing water, followed by Bubisa and Marsabit Central. On the other hand, households in Sagante/Jaldesa, Laisamis, and Logologo spent relatively less time fetching water from the various sources (Table 1).

Table 2 provides a summary of the percentage of households using different types of toilet facilities and fecal waste disposal methods. The study shows that the majority of households using pit latrines were from the Sagante/Jaldesa ward, followed by Marsabit Central, Laisamis, Karare, and Logologo. The Bubisa ward did not register any pit latrines; however, there were communal pit latrines constructed by development agencies. The lack of pit latrines was more prevalent in lowland wards (Laisamis, Karare, Logologo, Sagante/Jaldesa, and Bubisa) than in the urban ward in Marsabit Central. In such circumstances, many affected households (94.9%) resorted to open defecation, either in the open field or in the bush (*p* < .001). Only 5.1% of households without pit latrines preferred to use their neighbor's toilet facility. Open defecation was practiced more in pastoral lowland wards, such as Karare, Laisamis, Bubisa, Logologo, and Sagante/Jaldesa.

**TABLE 2:** Frequencies of population characteristics in different wards in Marsabit County.

Household characteristics	Name of ward						Total	*p* value
	Karare (*n* = 53)	Laisamis (*n* = 69)	Logologo (*n* = 43)	Central (*n* = 62)	Sagante/Jaldesa (*n* = 118)	Bubisa (n = 49)		
Household head's education								
Illiterate	71.7	60.9	79.1	24.2	42.4	63.3	53.3	<.001
Primary	11.3	15.9	4.7	41.9	35.6	16.3	24.1	
Postsecondary	17.0	23.2	16.3	33.9	22.0	20.4	22.6	
Household head's marital status								
Married	90.6	89.9	86.0	77.4	86.4	93.9	87.1	.163
Divorced	1.9	4.3	4.7	11.3	5.9	4.1	5.6	
Single	1.9	2.9	0.0	8.1	1.7	2.0	2.8	
Widowed	5.7	2.9	9.3	3.2	5.9	0.0	4.6	
Estimated monthly income (KES^†^)								
<5000	73.6	46.4	58.1	72.6	87.3	46.9	67.8	<.001
5001–10,000	22.6	31.9	30.2	8 12.9	7.6	28.6	19.8	
10,000–15,000	1 1.9	18.8	11.6	3 4.8	1.7	10.2	7.4	
15,001–20,000	0.0	0.0	0.0	3.2	3.4	6.1	2.3	
>20,000	1.9	2.9	0.0	6.5	0.0	8.2	2.8	
Caregiver's occupation								
Livestock	58.5	56.5	69.8	0.0	17.8	63.3	38.6	<.001
Labor	18.9	10.1	14.0	27.4	33.1	10.2	21.4	
Employed	7.5	13.0	4.7	17.7	13.6	8.2	11.7	
Self‐employed	3.8	11.6	2.3	43.5	20.3	8.2	16.8	
Pensioner	0.0	0.0	4.7	0.0	0.8	2.0	1.0	
Unemployed	11.3	8.7	4.7	11.3	14.4	8.2	10.7	
Caregiver's marital status								
Married	90.6	89.9	86.0	77.4	86.4	93.9	87.1	.163
Single	1.9	2.9	0.0	8.1	1.7	2.0	2.8	
Divorced	1.9	4.3	4.7	11.3	5.9	4.1	5.6	
Widowed	5.7	2.9	9.3	3.2	5.9	0.0	4.6	
Caregiver's education								
None	75.5	68.1	86	33.9	66.9	73.5	66.0	<.001
Primary	15.1	18.8	7.0	40.3	25.4	20.4	22.6	
>Secondary	9.4	13.0	7.0	25.8	7.6	6.1	11.4	
Sex of child								
Male	45.3	62.3	44.2	46.8	57.6	67.3	54.8	.06
Female	54.7	37.7	55.8	53.2	42.4	32.7	45.2	
Child's age category								
6–11	3.8^a^	13.0^ab^	18.6^ab^	22.6^ab^	15.3^ab^	24.5^b^	16.0	.39
12–17	7.5^a^	24.6^a^	18.6^a^	8.1^a^	11.9^a^	22.4^a^	15.0	
18–23	20.8^a^	15.5^a^	23.3^a^	24.2^a^	16.1^a^	14.3^a^	18.3	
24–35	34^a^	29^a^	30.2^a^	30.6^a^	29.7^a^	28.6^a^	30.2	
36–47	24.5^a^	15.9^a^	7.0^a^	9.7^a^	19.5^a^	8.2^a^	15.2	
48–59	9.4^a^	2.9^a^	2.3^a^	4.8^a^	7.6^a^	2.0^a^	5.3	
Vaccination								
Yes	88.7	82.6	86.0	96.8	94.1	98.0	91.4	.011
Diarrhea								
Yes	15.1	15.9	11.6	4.8	6.8	69.4	17.5	<.001
Ever breastfed								
Yes	100	100	97.7	100	100	100	99.5	.511
No access to toilet								
Yes	71.7	76.8	79.1	3.2	18.6	59.2	45.2	<.001
Open defecation								
Yes	100^a^	100^a^	88.2^a^	0.0^b^	100^a^	89.7^a^	94.9	<.001
Use neighbor's toilet								
Yes	0.0^a^	0.0^a^	11.8^a^	100^b^	0.0^a^	10.3^a^	5.1	<.001

*Note*: Values with the same letters are not significantly different at *p* < .05.

^†^ 1USD = KES 150.00.

### 3.3 Nutritional status of children aged 6–59 months in the study areas

**FIGURE 4:**
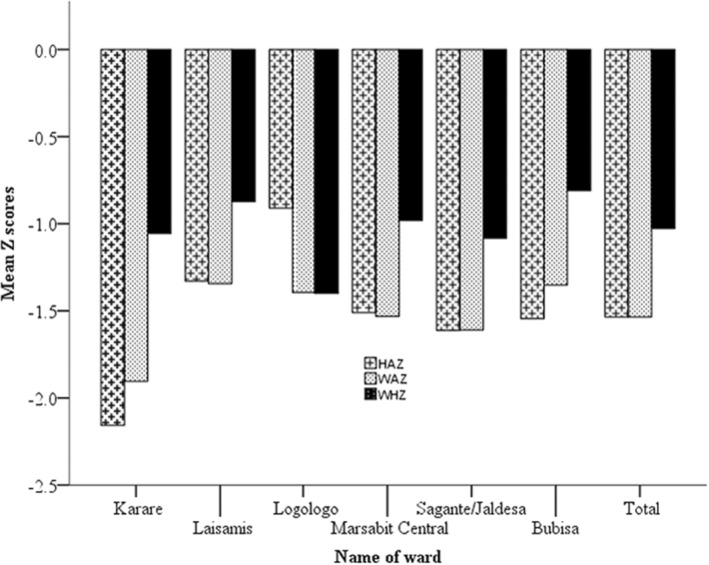
Mean nutritional status of children by ward (*N* = 394). Error bars represent the 95% confidence interval: *χ* ^2^; *p* < .001.

Figure 4 presents a summary of anthropometric Z‐scores for nutritional status indicators among children in Marsabit. The mean HAZ, WAZ, and WHZ were −1.51, 1.54, and 1.02, respectively. Children in the Logologo and Laisamis wards had significantly higher HAZ than those in the Central, Sagante/Jaldesa, and Bubisa wards. Regarding WHZ, there were no significant differences across the wards. The mean WHZs in the Karare, Laisamis, Logologo, Central, Sagante/Jaldesa, and Bubisa wards were −1.06, −0.87, −1.32, −0.98, −1.08, and −0.81, respectively.

Table 3 provides a summary of the prevalence of stunting, wasting, and underweight among children in the six wards, categorized by age groups. The study revealed significant (*p* < .001) differences in overall moderate (33%) and severe (3.8%) stunting across the wards. Karare had the highest prevalence of moderate to severe stunting (49%), followed by Marsabit Central (41%), Sagante/Jaldesa (35%), and Bubisa (29%). In terms of sex, males showed slightly better nutritional status than females, with 38% of males and 39% of females exhibiting moderate to severe stunting (Table 3).

**TABLE 3:** Prevalence of stunting, wasting, and underweight among children by age group and ward.

Age group		Name of wards						Total (*N* = 394)	*χ* ^2^	Sig.
		Karare (*n* = 53)	Laisamis (*n* = 69)	Logologo (*n* = 43)	Central (*n* = 62)	Sagante/Jaldesa (*n* = 118)	Bubisa (*n* = 49)			
	*Stunting*									
6–11	Normal	2 (100)^a^	9 (100)^a^	8 (100)^a^	13 (86.7)^a^	17 (94.4)^a^	9 (81.8)^a^	58 (92.1)	3.956	.556
	Moderate	0 (0.0)^a^	0 (0.0)^a^	0 (0.0)^a^	2 (13.3)^a^	1 (5.6)^a^	2 (18.2)^a^	5 (7.9)		
12–17	Normal	2 (66.7)^a^	17 (94.4)^a^	8 (88.9)^a^	4 (80.0)^a^	13 (86.7)^a^	12 (92.3)^a^	56 (88.9)	14.757	.141
	Moderate	1 (33.3)^a^	1 (5.6)^a^	1 (11.1)^a^	0 (0.0)^a^	2 (13.3)^a^	1 (7.7)^a^	6 (9.5)		
	Severe	0 (0.0)^a^	0 (0.0)^a^	0 (0.0)^a^	1 (20.0)^a^	0 (0.0)^a^	0 (0.0)^a^	1 (1.6)		
18–23	Normal	7 (50.0)^a^	2 (22.2)^a^	8 (88.9)^a^	10 (71.4)^a^	13 (68.4)^a^	4 (66.7)^a^	44 (62.0)	15.572	.113
	Moderate	7 (50.0)^a^	7 (77.8)^a^	1 (11.1)^a^	3 (21,4)^a^	6 (31.6)^a^	2 (33.3)^a^	26 (36.6)		
	Severe	0 (0.0)^a^	0 (0.0)^a^	0 (0.0)^a^	1 (7.1)^a^	0 (0.0)^a^	0 (0.0)^a^	1 (1.4)		
24–35	Normal	8 (47.1)^a^	6 (28.6)^a^	9 (69.2)^a^	7 (38.9)^a^	21 (58.3)^a^	7 (53.8)^a^	58 (49.2)	22.513	.013
	Moderate	9 (52.9)^a,b^	15 (71.4)^b^	1 (7.7)^a^	10 (55.6)^a,b^	13 (36.1)^a,b^	4 (30.8)^a,b^	52 (44.1)		
	Severe	0 (0.0)^a^	0 (0.0)^a^	3 (23.1)^a^	1 (5.6)^a^	2 (5.6)^a^	2 (15.4)^a^	8 (6.8)		
36–47	Normal	6 (46.2)^a^	1 (10.0)^a^	1 (33.3)^a^	1 (14.3)^a^	8 (36.4)^a^	2 (40.0)^a^	19 (31.7)	9.301	.504
	Moderate	5 (38.5)^a^	9 (90.0)^a^	2 (66.7)^a^	5 (71.4)^a^	13 (59.1)^a^	3 (60.0)^a^	37 (61.7)		
	Severe	2 (15.4)^a^	0 (0.0)^a^	0 (0.0)^a^	1 (14.3)^a^	1 (4.5)^a^	0 (0.0)^a^	4 (6.7)		
48–59	Normal	2 (50.0)^a^	0 (0.0)^a^	0 (0.0)^a^	1 (33.3)^a^	5 (62.5)^a^	1 (100)^a^	9 (47.4)	23.046	.011
	Moderate	2 (50.0)^a^	2 (100)^a^	0 (0.0)^a^	2 (66.7)^a^	3 (37.5)^a^	0 (0.0)^a^	9 (47.4)		
	Severe	0 (0.0)^a^	0 (0.0)^a,b^	1 (100)^b^	0 (0.0)^a,b^	0 (0.0)^a^	0 (0.0)^a,b^	1 (5.3)		
Total	Normal	27 (50.9)^a,b^	35 (50.7)^b^	34 (79.1)^a^	36 (58.1)^a,b^	77 (65.3)^a,b^	35 (71.4)^a,b^	244 (61.9)	27.614	.002
	Moderate	24 (45.3)^a^	34 (49.3)^a^	5 (11.6)^b^	22 (35.5)^a,b^	38 (32.2)^a,b^	12 (24.5)^a,b^	135 (34.3)		
	Severe	2 (3.8)^a^	0 (0.0)^a^	4 (9.3)^a^	4 (6.5)^a^	3 (2.5)^a^	2 (4.1)^a^	15 (3.8)		
	*Underweight*									
6–11	Normal	1 (50.0)^a^	8 (88.9)^a^	7 (87.5)^a^	9 (60.0)^a^	14 (77.8)^a^	8 (72.7)^a^	47 (74.6)	4.115	.533
	Moderate	1 (50.0)^a^	1 (11.1)^a^	1 (12.5)^a^	6 (40.0)^a^	4 (22.2)^a^	3 (27.3)^a^	16 (25.4)		
12–17	Normal	1 (33.3)^a^	17 (94.4)^a^	8 (88.9)^a^	3 (60.0)^a^	14 (93.3)^a^	9 (69.2)^a^	52 (82.5)	15.149	.127
	Moderate	2 (66.7)^a^	1 (5.6)^a^	1 (11.1)^a^	2 (40.0)^a^	1 (6.7)^a^	3 (23.1)^a^	10 (15.9)		
	Severe	0 (0.0)^a^	0 (0.0)^a^	0 (0.0)^a^	0 (0.0)^a^	0 (0.0)^a^	1 (7.7)^a^	1 (1.6)		
18–23	Normal	8 (57.1)^a^	6 (66.7)^a^	8 (88.9)^a^	11 (78.6)^a^	13 (68.4)^a^	5 (83.3)^a^	51 (71.8)	10.874	.367
	Moderate	6 (42.9)^a^	3 (33.3)^a^	0 (0.0)^a^	2 (14.3)^a^	6 (31.6)^a^	1 (16.7)^a^	18 (25.4)		
	Severe	0 (0.0)^a^	0 (0.0)^a^	1 (0.0)^a^	1 (7.1)^a^	0 (0.0)^a^	0 (0.0)^a^	2 (2.8)		
24–35	Normal	12 (70.6)^a^	17 (81.0)^a^	11 (84.6)^a^	16 (88.9)^a^	25 (69.4)^a^	11 (84.6)^a^	92 (78.0)	4.089	.537
	Moderate	5 (29.4)^a^	4 (19.0)^a^	2 (15.4)^a^	2 (11.1)^a^	11 (30.6)^a^	2 (15.4)^a^	26 (22.0)		
36–47	Normal	5 (38.5^a^	5 (50)^a^	2 (66.7)^a^	4 (57.1^a^	13 (59.1)^a^	5 (100)^a^	34 (56.7)	10.949	.361
	Moderate	8 (61.5)^a^	4 (40.0)^a^	1 (33.3)^a^	3 (42.9)^a^	9 (40.9)^a^	0 (0.0)^a^	25 (41.7)		
	Severe	0 (0.0)^a^	1 (10.0)^a^	0 (0.0)^a^	0 (0.0)^a^	0 (0.0)^a^	0 (0.0)^a^	1 (1.7)		
48–59	Normal	2 (50.0)^a^	2 (100)^a^	0 (0.0)^a^	3 (100)^a^	3 (37.5)^a^	1 (100)^a^	11 (57.9)	7.206	.206
	Moderate	2 (50.0)^a^	0 (0.0)^a^	1 (100)^a^	0 (100)^a^	5 (62.5)^a^	0 (0.0)^a^	8 (42.1)		
Total	Normal	29 (54.7)^a^	55 (79.7)^b^	36 (83.7)^b^	46 (74.2)^a,b^	82 (69.5)^a,b^	39 (79.6)^a,b^	287 (72.8)	20.719	.023
	Moderate	24 (45.3)^a^	13 (18.8)^b^	6 (14.0)^b^	15 (24.2)^a,b^	36 (30.5)^a,b^	9 (18.4)^a,b^	103 (26.1)		
	Severe	0 (0.0)^a^	1 (1.4)^a^	1 (2.3)^a^	1 (1.6)^a^	0 (0.0)^a^	1 (2.0)^a^	4 (1.0)		
	*Wasting*									
6–11	Normal	2 (100)^a^	7 (77.8)^a^	6 (75.0)^a^	11 (73.3)^a^	13 (72.2)^a^	8 (72.7)_a_	47 (74.6)	2.689	.988
	Moderate	0 (0.0)^a^	2 (22.2)^a^	2 (25.0)^a^	3 (20.0)^a^	4 (22.2)^a^	3 (27.3)_a_	14 (22.2)		
	Severe	0 (0.0)^a^	0 (0.0)^a^	0 (0.0)^a^	1 (6.7)^a^	1 (5.6)^a^	0 (0.0)_a_	2 (3.2)		
12–17	Normal	3 (100)^a^	17 (94.4)^a^	8 (88.9)^a^	4 (80.0)^a^	12 (80.0)^a^	10 (76.9)_a_	54 (85.7)	6.317	.788
	Moderate	0 (0.0)^a^	1 (5.6)^a^	1 (11.1)^a^	1 (20.0)^a^	3 (20.0)^a^	2 (15.4)_a_	8 (12.7)		
	Severe	0 (0.0)^a^	0 (0.0)^a^	0 (0.0)^a^	0 (0.0)^a^	0 (0.0)^a^	1 (7.7)_a_	1 (1.6)		
18–23	Normal	12 (85.7)^a^	8 (88.9)^a^	3 (33.3)^a^	8 (57.1)^a^	14 (73.7)^a^	6 (100)_a_	51 (71.8)	17.21	.070
	Moderate	2 (14.3)^a^	1 (11.1)^a^	4 (44.4)^a^	4 (28.6)^a^	5 (26.3)^a^	0 (0.0)_a_	16 (22.5)		
	Severe	0 (0.0)^a^	0 (0.0)^a^	2 (22.2)^a^	2 (14.3)^a^	0 (0.0)^a^	0 (0.0)_a_	4 (5.6)		
24–35	Normal	15 (88.2)^a^	19 (90.5)^a^	10 (76.9)^a^	18 (100.0)^a^	27 (75.0)^a^	10 (76.9)_a_	99 (83.9)	8.993	.533
	Moderate	2 (11.8)^a^	2 (9.5)^a^	3 (23.1)^a^	0 (0.0)^a^	8 (22.2)^a^	3 (23.1)^a^	18 (15.3)		
	Severe	0 (0.0)^a^	0 (0.0)^a^	0 (0.0)^a^	0 (0.0)^a^	1 (2.8)^a^	0 (0.0)^a^	1 (0.8)		
36–47	Normal	10 (76.9)^a^	9 (90.0)^a^	3 (100)^a^	6 (85.7)^a^	20 (90.9)^a^	5 (100)^a^	53 (88.3)	5.279	.872
	Moderate	2 (15.4)^a^	1 (10.0)^a^	0 (0.0)^a^	1 (14.3)^a^	2 (9.1)^a^	0 (0.0)^a^	6 (10.0)		
	Severe	1 (7.7)^a^	0 (0.0)^a^	0 (0.0)^a^	0 (0.0)^a^	0 (0.0)^a^	0 (0.0)^a^	1 (1.7)		
48–59	Normal	3 (75.0)^a^	2 (100)^a^	1 (100)^a^	3 (100)^a^	7 (100)^a^	1 (100)^a^	17 (89.5)	1.746	.883
	Moderate	1 (25.0)^a^	0 (0.0)^a^	0 (0.0)^a^	0 (0.0)^a^	1 (12.5)^a^	0 (0.0)^a^	2 (10.5)		
Total	Normal	45 (84.9)^a^	62 (89.9)^a^	31 (72.1)^a^	50 (80.6)^a^	93 (78.8)^a^	40 (81.6)	321 (81.5)	9.937	.446
	Moderate	7 (13.2)^a^	7 (10.1)^a^	10 (23.3)^a^	9 (14.5)^a^	23 (19.5)^a^	8 (16.3)	64 (16.2)		
	Severe	1 (1.9)^a^	0 (0.0)^a^	2 (4.7)^a^	3 (4.8)^a^	2 (1.7)^a^	1 (2.0)	9 (2.3)		

*Note*: Values with the same letters are not significantly different at *p* < .05.

Underweight differed significantly across the wards (*p* = .023), with 73% of children within the normal weight range, 26% moderately underweight, and 1.0% severely underweight (Table 3). Karare had the highest prevalence of underweight among children aged 6–59 months, followed by Sagante/Jaldesa, Marsabit Central, Logologo, and Bubisa (Table 3). In terms of sex, 27% of males and 25% of females were moderately underweight, while 0.5% of males and 2.0% of females were severely underweight. Moderate to severe underweight among female children ranged from 19% in Logologo to 35% in Laisamis, while among male children, it ranged from 10.5% in Logologo to 58.3% in Karare.

The overall wasting prevalence was 16%, with 2% severely wasted (*p* > .05). Prevalence varied by age, the highest among 18–23 months (20%), followed by 24–35 months (19%), and 6–11 months (16%). Wardwise, Logologo had the highest prevalence among females (25%), while Sagante/Jaldesa had the highest prevalence among males (32%; Table 3).

### 3.4 Social, economic, demographic, and environmental factors influencing undernutrition among children

Table 4 outlines the factors influencing stunting, underweight, and wasting among pastoral children in northern Kenya. Significant predictors of stunting included the child's age, drinking water source, and caregiver's occupation. Younger children within 1000 days were more likely to experience stunting (COR = 1.078, 95% CI: 1.056–1.099, *p* < .001). Moreover, children from households using water from public pans (AOR = 3.704, 95% CI: 1.594–8.608, *p* = .002) or unprotected wells (AOR = 3.131, 95% CI: 1.045–9.380, *p* = .042) were at a higher risk of stunting. Conversely, children with unemployed caregivers were less likely to be stunted (AOR = 0.603, 95% CI: 0.437–0.832, *p* = .002). In the study area, children who belonged to households with inadequate access to toilet facilities had a 3.8‐fold increased likelihood of being stunted (AOR = 3.797, 95% CI: 1.863–7.737, *p* < .001). Additionally, appropriate disposal of child fecal waste was associated with a significantly lower likelihood of stunting (AOR = 0.257, 95% CI: 0.102–0.645, *p* = .004). A greater distance to the nearest market center was significantly associated with an increased likelihood of stunting in children in Karare ward by a factor of 1.2 (AOR = 1.151, 95% CI: 1.031–1.285, *p* = .012). The age of the child was likely to significantly affect stunting in the Sagante/Jaldesa (AOR = 1.088, 95% CI: 1.026–1.153, *p* = .005) and Bubisa (AOR = 1.116, 95% CI:1.029–1.210, *p* = .008) wards, respectively (Table 5).

**TABLE 4:** Overall odds ratio (OR) of factors associated with undernutrition among children 6–59 months in Marsabit County.

Predictors	Stunting (HAZ <‐2SD)				Underweight (WAZ <‐2SD)				Wasting (WHZ <‐2SD)			
	COR (95% CI)	*p* value	AOR (95% CI)	*p* value	COR (95% CI)	*p* value	AOR (95% CI)	*p* value	COR (95% CI)	*p* value	AOR (95% CI)	*p* value
HH's education												
None	Ref	.086	1.125 (0.45, 2.17)	.793	0.544 (0.32, 0.93)	.025*	0.582 (0.19, 1.79)	.345	0.478 (0.25, 0.90)	.023*	0.287 (0.10, 0.87)	.027*
Primary	0.641 (0.39,1.07)											
HH's occupation												
Livestock keeping	Ref											
Labor/herder	0.49 (0.24,0.99)	.045*	0.475 (0.18, 1.26)	.135	0.508 (0.24, 1.08)	.078	1.523 (0.39, 5.99)	.547	0.840 (0.33, 2.13)	.712	2.278 (0.63, 8.28)	.211
Child's age (mean)	1.078 (1.056, 1.099)	.000	1.082 (0.98, 1.19)	.111	1.025 (1.01, 1.04)	.006	1.844 (1.50, 2.26)	.000	0.975 (0.95, 0.10)	.032	1.341 (1.16, 1.55)	.000
Grouped child's age												
6–11	Ref	.000		0.002		.015		.175		.087		.063
12–17	0.051 (0.01, 0.19)	.000	0.023 (0.00, 1.31)	.067	0.407 (0.15,1.13)	.084	0.182 (0.00, 74.23)	.578	3.511 (0.74, 16.70)	.115	21.852 (0.10, 4901.11)	.264
18–23	0.118 (0.038, 0.368)	.000	0.099 (0.00, 3.38)	.200	0.224 (0.08, 0.67)	.007	0.939 (0.00, 213.20)	.982	1.279 (0.24, 6.71)	.771	24.005 (0.179, 3224.81	.204
24–35	0.375 (0.14, 1.01)	.053	0.3 (0.02, 5.88)	.428	0.367 (0.134, 1.006)	.051	0.762 (0.008, 75.218)	.908	2.293 (0.477, 11.017)	.300	23.422 (0.363, 1510.647)	.138
36–47	0.69 (0.27, 1.78)	.436	0.708 (0.07, 7.24)	.771	0.338 (0.13, 0.88)	.026	0.827 (0.02, 30.95)	.918	1.805 (0.39, 8.40)	.452	19.94 (0.72, 551.10)	.077
Drinking water source												
Borehole	Ref	.023		.000		.032		.174		.882		.723
Public pan	1.468 (0.82, 2.63)	.197	3.704 (1,59, 8.61)	.002	0.453 (0.25, 0.83)	.01	0.318 (0.11, 0.93)	.035	0.731 (0.36, 1.47)	.381	0.844 (0.32, 2.23)	.732
Unprotected well	1.346 (0.595, 3.046)	.475	3.131 (1.05, 9.38)	.042	0.538 (0.22, 1.29)	.166	0.458 (0.11, 1.84)	.271	0.590 (0.20, 1.76)	.345	0.438 (0.10, 1.88)	.267
Access to toilet facility	1.783 (1.173, 2.712)	.007	3.797 (1.86, 7.74)	.000	1.503 (0.96, 2.36)	.078	1.175 (0.47, 2.97)	.733	1.071 (0.63, 1.84)	.802	0.948 (0.42, 2.14)	.898
Fecal waste disposal	0.660 (0.36, 1.21)	.181	0.257 (0.10, 0.65)	.004	1.152 (0.58, 2.31)	.69	0.719 (0.21, 2.49)	.603	0.531 (0.26, 1.09)	.083	0.347 (0.12, 1.02)	.054
Caregiver's income												
< 5000 per month	Ref	.841	1.086 (0.48, 2.47)	.844		.044		.04		.695		.63
5001–10,000	0.730 (0.217, 2.454)	.611	0.444 (0.17, 1.19)	.105	0.232 (0.07, 0.81)	.023	0.036 (0.00, 0.47)	.011	0.888 (0.19, 4.25)	.882	2.002 (0.17, 23.87)	.583
10,000–15,000	0.635 (0.177, 2.274)	.486	0.829 (0.12, 5.94)	.852	0.125 (0.03, 0.49)	.003	0.017 (0.00, 0.26)	.003	0.739 (0.14, 3.88)	.721	2.124 (0.16, 27.98	.567
15,001–20,000	0.733 (0.180, 2.986)	.665	1.460 (0.24, 8.81)	.68	0.218 (0.05, 0.95)	.043	0.053 (0.00, 0.90)	.042	0.720 (0.11, 4.63)	.729	1.927 (0.14, 27.03)	.626
>20,000	0.343 (0.048, 2.457)	.287	0.929 (0.39, 2.21)	.868	0.163 (0.02, 1.20)	.075	0.018 (0.00, 0.62)	.026	2.245 (0.29, 17.76)	.442	6.842 (0.54, 86.31)	.137
Child's weight	1.419 (1.257, 1.603)	.000	1.000 (1.00, 1.00)	.395	0.835 (0.74, 0.94)	.004	0.013 (0.01, 0.04)	.000	0.661 (0.56, 0.78)	.000	0.128 (0.07, 0.24)	.000
Caregiver's occupation												
Unemployed	1.615 (0.87, 3.01)	.131	0.603 (0.44, 0.83)	.002	1.079 (0.55, 2.12)	.826	0.763 (0.25, 2.34)	.635	1.098 (0.49, 2.47)	.822	1.036 (0.36, 3.01)	.949

*Note*: *p < .05.

**TABLE 5:** Significant household factors influencing stunting, underweight, and wasting by ward.

Ward	Predictor variables	AOR (95% CI)	*p* value
		*Stunting*	
Karare	Distance to market	1.151 (1.031, 1.285)	.012
Sagante/Jaldesa	Child's age in months	1.088 (1.026, 1.153)	.005
Bubisa	Child's age in months	1.116 (1.029, 1.210)	.008
		*Underweight*	
Sagante/Jaldesa	Family size	0.091 (0.021, 0.393)	.001
	Age of caregiver (years)	1.447 (1.125, 1.860)	.004
Bubisa	Caregiver's monthly income	1.000 (1.000, 1.000)	.049
	Weight (kg)	0.517 (0.290, 0.923)	.026
		*Wasting*	
Laisamis	Weight (kg)	0.066 (0.004, 0.979)	.048
Sagante/Jaldesa	Caregiver's monthly income	1.000 (1.000, 1.000)	.039
	Child's age (months)	1.370 (1.009, 1.861)	.044
	Weight (kg)	0.046 (0.003, 0.706)	.027
	Wash hands before feeding child	0.058 (0.002, 1.531)	.088
Bubisa	Family size	0.216 (0.061, 0.770)	.018

The main predictors of underweight included household head's highest level of education, age of the child, source of drinking water, caregiver's income, and child's weight, respectively (Table 4). Children residing in households where the household head attained at least primary education exhibited a reduced likelihood of being underweight (COR = 0.544, 95% CI: 0.319–0.928, *p* = .025). Furthermore, the age of the child emerged as a significant factor associated with an increased probability of underweight (AOR = 1.844, 95% CI: 1.502–2.264, *p* < .001). Notably, children from households accessing drinking water from public water pans demonstrated a decreased likelihood of experiencing underweight conditions (AOR = 0.318, 95% CI: 0.109–0.925, *p* = .035). Furthermore, higher caregiver income was associated with a lower likelihood of child underweight (AOR = 0.036, 95% CI: 0.003–0.467, *p* < .05). The child's weight (AOR = 0.013, 95% CI: 0.005–0.037, *p* < .001) demonstrated a reducing effect on underweight. Additionally, every unit increase in age reduced underweight by 0.031 units (Table 4).

In Sagante/Jaldesa, a smaller family size was linked to a lower likelihood of underweight (AOR = 0.091, 95% CI: 0.021–0.393, *p* = .001), while the caregiver's age showed an increased risk of child underweight (AOR = 1.447, 95% CI: 1.125–1.860, *p* = .004). Conversely, in Bubisa ward, low caregiver monthly income was associated with a higher likelihood of child underweight (AOR = 1.000, 95% CI: 1.000–1.000, *p* = .049), while a greater child weight was linked to reduced odds of child underweight (AOR = 0.517–95% CI: 0.290–0.923, *p* = .026; Table 5).

The results indicate significant associations between certain factors and the likelihood of wasting among children. Specifically, the age of the child showed a notable increase in the likelihood of wasting (AOR = 31.34, 95% CI: 1.160–1.551, *p* < .001). Conversely, weight (AOR = 0.128, 95% CI: 0.069–0.239, *p* < .001), household head's education (AOR = 0.287, 95% CI: 0.095–0.868, *p* = .027), and safe disposal of child fecal waste (AOR = 0.274, 95% CI: 0.118–1.017, *p* = .054) were associated with a reduced likelihood of wasting.

At the ward level, the results reveal that in the Laisamis ward, the weight of the child emerged as the sole significant predictor of wasting (AOR = 0.066, 95% CI: 0.004–0.979, *p* = .048). Conversely, in the Sagante/Jaldesa ward, caregiver's monthly income (AOR = 1.000, 95% CI: 1.00–1.00, *p* = .014) and child's age (AOR = 1.370, 95% C.: 1.009–1.861, *p* = .044) were found to increase the likelihood of wasting. Additionally, weight (AOR = 0.046, 95% CI: 0.003–0.706, *p* = .027) emerged as a major significant factor in the Sagante/Jaldesa ward. Furthermore, family size (AOR = 0.216, 95% CI: 0.061–0.770, *p* = .018) was predictive of wasting in the Bubisa ward (Table 5).

## 4 DISCUSSION

The levels of underweight, stunting, and wasting described in the current study (Tables 3 and 6) exceed the WHO recommended cutoff values of <10% for underweight, <20% for stunting, and <5% for wasting (de Onis et al., 2019; WHO, 2006). Furthermore, the prevalence of stunting, wasting, and underweight was higher in comparison to that mentioned in the Kenya Demographic and Health Surveys (KNBS and ICF, 2023; KNBS, MOH/Kenya, 2015). A high undernutrition rate may be attributed to inadequate dietary intakes and poor digestion arising from child morbidity, hence resulting in poor muscle mass development and cumulative linear growth retardation.

**TABLE 6:** Prevalence of stunting, wasting, and underweight among children by sex and ward.

Sex	Indicators	Name of wards						*N* = 394	*χ* ^2^; *p* ≤ .05
	status	Karare (*n* = 53)	Laisamis (*n* = 69)	Logologo (*n* = 43)	Central (*n* = 62)	Sagante/Jaldesa (*n* = 118)	Bubisa (*n* = 49)		
Stunting, *n* (%)									
Male	Normal	15 (62.5)	22 (51.2)	13 (68.4)	13 (44.8)	47 (69.1)	25 (75.8)	135 (62.5)	.01
	Moderate	29.2)	21 (48.8)	3 (15.8)	14 (48.3)	20 (29.4)	6 (18.2)	71 (32.9)	
	Severe	2 (8.3)	0 (0.0)	3 (15.8)	2 (6.9)	1 (1.5)	2 (6.1)	10 (4.6)	
Female	Normal	12 (41.4)	13 (50)	21 (87.5)	23 (69.7)	30 (60)	10 (62.5)	109 (61.2)	.02
	Moderate	17 (58.6)	13 (50)	2 (8.3)	8 (24.2)	18 (36)	6 (37.5)	64 (36.0)	
	Severe	0 (0.0)	0 (0.0)	1 (4.2)	2 (6.1)	2 (4.0)	0 (0.0)	5 (2.8)	
Total	Normal	27 (50.9)	35 (50.7)	34 (79.1)	36 (58.1)	77 (65.3)	35 (71.4)	244 (61.9)	.002
	Moderate	24 (45.3)	34 (49.3)	5 (11.6)	22 (35.5)	38 (32.2)	12 (24.5)	135 (34.3)	
	Severe	2 (3.8)	0 (0.0)	4 (9.3)	4 (6.5)	3 (2.5)	2 (4.1)	15 (3.8)	
Underweight, *n* (%)									
Male	Normal	10 (41.7)	38 (88.4)	17 (89.5)	21 (72.4)	45 (66.2)	26 (78.8)	157 (72.7)	.001
	Moderate	14 (58.3)	5 (11.6)	2 (10.5)	7 (24.1)	23 (33.8)	7 (21.2)	58 (26.9)	
	Severe	0 (0.0)	0 (0.0)	0 (0.0)	1 (3.4)	0 (0.0)	0 (0.0)	1 (0.5)	
Female	Normal	19 (65.5)	17 (65.4)	19 (79.2)	25 (75.8)	37 (74)	13 (81.2)	130 (73)	.509
	Moderate	10 (34.5)	8 (30.8)	4 (16.7)	8 (24.2)	13 (26.0)	2 (12.5)	45 (25.3)	
	Severe	0 (0.0)	1 (3.8)	1 (4.2)	0 (0.0)	0 (0.0)	1 (6.2)	3 (1.7)	
Total	Normal	29 (54.7)	55 (79.7)	36 (83.7)	46 (74.2)	82 (69.5)	39 (79.6)	287 (72.8)	.023
	Moderate	24 (45.3)	13 (18.8)	6 (14.0)	15 (24.2)	36 (30.5)	9 (18.4)	103 (26.1)	
	Severe	0 (0.0)	1 (1.4)	1 (2.3)	1 (1.6)	0 (0.0)	1 (2.0)	4 (1.0)	
Wasting, *n* (%)									
Male	Normal	20 (83.3)	38 (88.4)	13 (68.4)	24 (82.8)	54 (79.4)	27 (81.8)	176 (81.5)	.716
	Moderate	4 (16.7)	5 (11.6)	5 (26.3)	4 (13.8)	13 (19.1)	6 (18.2)	37 (17.1)	
	Severe	0 (0.0)	0 (0.0)	1 (5.3)	1 (3.4)	1 (1.5)	0 (0.0)	3 (1.4)	
Female	Normal	25 (86.2)	24 (92.3)	18 (75)	26 (78.8)	39 (78)	13 (81.2)	145 (81.5)	.837
	Moderate	3 (10.3)	2 (7.7)	5 (20.8)	5 (15.2)	10 (20.0)	2 (12.5)	27 (15.2)	
	Severe	1 (3.4)	0 (0.0)	1 (4.2)	2 (6.1)	1 (2.0)	1 (6.2)	6 (3.4)	
Total	Normal	45 (84.9)	62 (89.9)	31 (72.1)	50 (80.6)	93 (78.8)	40 (81.6)	321 (81.5)	.446
	Moderate	7 (13.2)	7 (10.1)	10 (23.3)	9 (14.5)	23 (19.5)	8 (16.3)	64 (16.2)	
	Severe	1 (1.9)	0 (0.0)	2 (4.7)	3 (4.8)	2 (1.7)	1 (2.0)	9 (2.3)	

*Note*: Values in parentheses are the percentages, and those outside are frequencies.

Other studies conducted in different regions have reported varying levels of undernutrition. For instance, Okidi et al. (2022) reported a higher prevalence of stunting (48.3%–58.4%), underweight (46%–54%), and wasting (36.4%–52.1%) among pastroral children in Karamoja in Uganda, whereas Khan et al. (2019) found high rates of stunting (44.4%), underweight (29.4%), and wasting (10.7%), respectively, among children aged 6–59 months in Pakistan. Furthermore, comparing the findings of the current study to those of the research works conducted in Uganda (Nankinga et al., 2019) and Senegal (Badiane et al., 2021), the prevalence rates of undernutrition were found to be higher in the present study. Conversely, Ma'alin et al. (2016) reported lower prevalence rates of underweight (24.5%), stunting (33.4%), and wasting (20%) in Shinile Woreda, Ethiopia. Similarly, Poudel et al. (2022) documented relatively lower rates of stunting (26.7%), wasting (7%), and undernutrition (17.7%) in the rural Kaski District, Nepal. The difference in prevalence rates may be attributed to differences in household's socioeconomic statuses and child feeding practices by caregivers.

Understanding the variations in undernutrition prevalence across different regions is vital for the development of targeted interventions and policies to address the specific challenges faced by each population. This highlights the need for context‐specific approaches that consider the unique socioeconomic, cultural, and environmental factors contributing to undernutrition. Moreover, these findings emphasize the importance of ongoing monitoring and evaluation of nutrition interventions to ensure their effectiveness in combating undernutrition among settled pastoral children in northern Kenya.

In the present study, children relying on public water pans or unprotected wells for drinking water exhibited a higher likelihood of stunting compared to those with access to safer water sources. These open water bodies in arid environments are susceptible to contamination from frequent sandstorms, potentially exposing children to diarrheal bacteria. Furthermore, limited access to toilets in the study areas was associated with increased stunting. This may contribute to open defecation, further contaminating the environment with harmful enteric pathogens. Children consuming water contaminated with these bacteria are at greater risk of contracting diarrheal diseases, known to impede growth and contribute to stunting. The notable diarrheal episodes in Bubisa ward can be explained by the fact that Bubisa is based in Chalbi desert and is prone to frequent sandstorms and open defecation is more common in the area. This may present with poor and dusty environmental conditions that predispose children to diarrhea‐causing foodborne microorganisms.

Numerous studies have investigated the association between stunting and various caregiver, child, and environmental factors (Ireri et al., 2021; Adeyonu et al., 2022; Mbijiwe et al., 2022). The consumption of contaminated water and lack of access to toilets can expose children to enteric diarrheal microorganisms, such as *Escherichia coli* and *Salmonella* spp., which are often associated with consuming contaminated food and water (Okidi et al., 2022). These findings here align with those of previous studies conducted among nonpastoral children in South Nikuman District, Cambodia (Blaney et al., 2019) and Indonesia (Lukman et al., 2022), pastoral households in the Karamoja region (Okidi et al., 2022), rural areas of North Sudan (Sulaiman et al., 2018), pastoral communities in Mieso‐Mulu District, Somali Regional State, Eastern Ethiopia (Geletaw et al., 2021). Furthermore, the findings in the current study compare well with those of Nsubuga et al. (2022) who reported an association between stunting and diarrhea among children in Uganda, and were also consistent with the findings of Sinha et al. (2018) among children in India.

Among pastoral households, common pathways for microbial contamination of infant complementary foods may include the use of raw or poorly cooked animal source foods. Enteric infection by foodborne pathogens can damage the mucosal layer of the intestine, followed by the activation of *eae* and other genes, causing dissolution of the normal microvilli structure. The bacterium then binds closely to the epithelial membrane via the protein *intimin* causing death of the epithelial cells and loss of the microvilli of the intestine. This results in the loss of nutrients and subsequent wasting and physical growth retardation (Forshell & Wierup, 2006).

These findings emphasize the importance of addressing access to safe drinking water, access to toilet facilities, caregiver's income, and child's weight in tackling stunting among children in northern Kenya. Moreover, findings here highlight the poor waste disposal methods where the majority of households use open defecation and use of poorly ventilated temporary pit latrines, particularly in lowland wards where the prevalence of households without toilets is higher. Access to well‐ventilated improved pit latrines in rural areas and use of flushable modern toilets in urban centers is crucial in promoting hygiene, preventing the spread of diseases, and improving the overall community health.

The latest research also revealed that unemployment among mothers, households led by wage earners, and the age of the child had a decreasing impact on stunting. This suggests that mothers who dedicate more time to caring for their children are less likely to witness child stunting. A study conducted in Dhaka, Bangladesh also reported that children of working mothers had nearly twice the odds of being stunted than children of nonworking mothers (Win et al., 2022). Nevertheless, multiple studies have indicated that children whose parents are employed are less susceptible to stunting (Ahmed et al., 2022; Yani et al., 2022). Additionally, income generated from labor‐related activities may influence food accessibility, leading to improved food security and consequently reduced stunting among children. The high rates of stunting within the age brackets of 24–35 months and 48–59 months witnessed in the current study may be attributed to short gestation spacing that gives preference to younger children and early weaning using poorly formulated complementary foods among pastoral communities. The results from the current study are consistent with findings from another study by Kang et al. (2018) which unraveled the correlation of stunting with age in Bhutan. Conversely, a study conducted among Karamoja children demonstrated that children aged 12–23 months, 36–47 months, and 48–59 months had reduced odds of being concurrently wasted and stunted with increasing age (Obeng‐Amoako et al., 2021). This phenomenon may be attributed to poor initiation of complementary feeding practices among pastoral households in Marsabit (Galgallo, 2017).

The association between distance to the nearest market center and stunting in the Karare and Laisamis wards was also observed (Table 5). Access to nearby markets can indirectly affect the nutritional status of young children through inability to regularly access fresh and nutritious foods sourced from food markets, technology adoption, employment opportunities, and the prices of agricultural products (Agyei‐Boakye, 2022; Weatherspoon et al., 2019).

Overall, the predictor model highlighted the age of the index child as a significant factor influencing stunting, wasting, and underweight across the wards. The higher stunting in Laisamis compared to the other wards may be attributed to drought conditions that prevailed at the time of conducting this study (Table 2). Settled households with children under the age of 5 faced significant limitations in accessing livestock products. Either the livestock was taken far in search for pasture and water or they would have died due to lack of feeds and water. The Rendille community, who live in this ward, primarily rely on livestock as their main source of nutrition and income generation. Despite heavy food aid through humanitarian support in pastoral areas, global acute malnutrition remains high and may be perpetuated by endemic food insecurity among settled households.

The current study also indicates that advancement in the mean age of child increased the likelihood of child being underweight by a factor of 1.8. This may be attributed to food insecurity and poor weaning practices that tend to focus on infants as compared to older children in the family (Wolde et al., 2015). This study was conducted during dry season when caregivers could not access milk from livestock. On the other hand, education of household head, safe disposal of fecal waste, and weight of the child showed reduced the likelihood of a child being underweight.

In the Sagante/Jaldesa ward, our study revealed a significant correlation between the age of caregivers and the prevalence of underweight children under the age of 5. Younger caregivers were notably associated with a heightened likelihood of undernutrition. Notably, the minimum age observed among caregivers was 16 years (not reported in the table), indicating the occurrence of early marriages among pastoral girls, which could potentially lead to teenage motherhood and subsequently poor feeding practices, thereby contributing to a higher incidence of undernutrition among children. These findings are consistent with those of prior research by Mbijiwe et al. (2022) and Morakinyo et al. (2020), which similarly linked younger caregivers with undernutrition in young children.

With a mean caregiver age of 28 years, it is evident that a significant proportion of children in this ward are at an elevated risk of undernutrition. This risk may be exacerbated by the high levels of illiteracy observed among households, reaching 66%. Previous studies have shown that the educational levels of caregivers have a notable impact on childhood undernutrition (Clarke et al., 2021; Paul & Saha, 2022; Stamenkovic et al., 2016). Education plays a pivotal role in empowering women to access essential healthcare information, thereby promoting better nourishment for their children (Iftikhar et al., 2017). Furthermore, improved education enhances women's prospects for securing decent employment and income, which in turn afford them greater autonomy in decision‐making regarding food choices for their children (Adeyonu et al., 2022; Iftikhar et al., 2017).

Moreover, the present study has revealed that family size exerts a significant influence on underweight in the Sagante/Jaldesa ward and wasting in the Bubisa ward. Family size directly impacts the allocation of food resources within households, with younger children typically receiving priority over those aged two and above. These findings are consistent with a prior investigation conducted in Ethiopia by Abera et al. (2019), which identified a notable correlation between larger family sizes and the prevalence of undernutrition among nonpastoral children under 5 years in Tigray, Ethiopia. Similarly, research conducted by Ahmad et al. (2019) in Pakistan demonstrated a significant link between family size and various indicators of undernutrition. Their findings indicated that family sizes ranging from 1 to 5 members had a mitigating effect on stunting and wasting.

Furthermore, caregiver's monthly income was found to significantly impact wasting and underweight in the Sagante/Jaldesa ward, while only predicting underweight in the Bubisa ward. Higher income levels afford caregivers the opportunity to make informed decisions regarding food purchases, thereby enhancing dietary diversity, particularly for complementary foods. Notably, in this study, 69% of caregivers were classified as low‐income earners, with a monthly income of $33 USD (see Table 1).

## 5 CONCLUSION

This study has shown that the prevalence of stunting, wasting, and undernutrition is above WHO standards. The main predictors of undernutrition included the age and weight of the index child, source of drinking water, waste disposal methods, and caregiver's characteristics, such as age, income, education, and family size. Development agencies need to focus on the supply of potable water, access to toilet facilities in addition to nutrition education on hygienic complementary feeding practices, and regular visits to health facilities to monitor the child's nutritional status should be encouraged among the pastoralists in Kenya.

## AUTHOR CONTRIBUTIONS

**Amos Otieno Adongo:** Conceptualization (lead); data curation (lead); formal analysis (lead); funding acquisition (lead); investigation (lead); methodology (equal); validation (equal); writing – original draft (lead); writing – review and editing (equal). **Joseph Wafula Matofari:** Conceptualization (equal); data curation (supporting); funding acquisition (supporting); investigation (supporting); methodology (equal); supervision (equal); writing – original draft (supporting); writing – review and editing (supporting). **Elizabeth Kamau Mbuthia:** Conceptualization (equal); data curation (supporting); methodology (supporting); supervision (supporting); writing – original draft (supporting); writing – review and editing (supporting).

## FUNDING INFORMATION

The study was funded by Egerton University, Njoro through the World Bank‐funded Centre of Excellence in Sustainable Agriculture and Agribusiness Management (CESAAM) scholarship.

## CONFLICT OF INTEREST STATEMENT

The authors declare no conflict of interest.

## ETHICS STATEMENT

This study was conducted according to the guidelines laid down in the Declaration of Helsinki and all procedures involving research study participants were approved by the Egerton University Review and Ethics Committee (approval no. EUREC/APP/088/2019). A research permit was acquired from the National Commission for Science, Technology and Innovations (NACOSTI) in Nairobi (license no. NACOSTI/P/20/2972). Written/verbal consent was obtained from child caregiver/mother before nutrition data were obtained. To ensure adherence to COVID‐19 protocols, data were collected with support from trained Ministry of Health officers distributed in the health facilities in each ward.

## Data Availability

The data that support the findings of this study are available on request from the corresponding author. The data are not publicly available due to privacy or ethical restrictions.

## References

[fsn34201-bib-0001] Abera, F. S. , Kantelhardt, E. J. , Bezabih, A. M. , Gebru, A. A. , Ejeta, G. , Lauvaia, J. , Wienke, A. , & Scherbaum, V. (2019). Nutrition‐specific and sensitive drivers of poor child nutrition in Kilte Awlaelo‐health and demographic surveillance site, Tigray, northern Ethiopia: Implications for public health nutrition in resource‐poor settings. Global Health Action, 12, 1556572. 10.1080/16549716.2018.1556572 31154991 PMC6338276

[fsn34201-bib-0002] Adeyonu, A. G. , Obisesan, A. A. , & Balogun, O. L. (2022). Determinants of malnutrition of under‐five children among rural households in the southwest, Nigeria. Food Research, 6, 215–222. 10.26656/fr.2017.6(1)729

[fsn34201-bib-0003] Agyei‐Boakye, O. P. (2022). Does proximity to markets affect child nutritional status? Evidence from Tanzania. doi:10.2139/ssrn.4089229 https://ssrn.com/abstract=4089229

[fsn34201-bib-0004] Ahmad, D. , Afzal, M. , & Imtiaz, A. (2019). Effect of socioeconomic factors on malnutrition among children in Pakistan. Future Business Journal, 6(1), 30. 10.1186/s43093-020-00032-x

[fsn34201-bib-0005] Ahmed, M. , Zepre, K. , Lentero, K. , Gebremariam, T. , Jemal, Z. , Wondimu, A. , Bedewi, J. , Melis, T. , & Gebremeskel, A. (2022). The relationship between maternal employment and stunting among 6–59 months old children in Gurage zone southern nation nationality People's region, Ethiopia: A comparative cross‐sectional study. Frontiers in Nutrition, 9, 964124. 10.3389/fnut.2022.964124 36276826 PMC9582235

[fsn34201-bib-0006] Asiimwe, R. , Ainembabazi, J. H. , Egeru, A. , Isoto, R. , Aleper, D. K. , Namaalwa, J. , & Diiro, G. M. (2020). The role of camel production on household resilience to droughts in pastoral and agro‐pastoral households in Uganda. Pastoralism: Research, Policy and Practice, 10, 5. 10.1186/s13570-020-0160-x

[fsn34201-bib-0007] Badiane, A. , Diouf, A. , Sylla, P. D. D. , Cisse, N. S. , Idohou‐Dossou, N. , Dramaix, M. , Wade, S. , & Donnen, P. (2021). Body composition and determinant factors among mother–child pairs (6–8 months) in rural areas of Senegal. Maternal & Child Nutrition, 2021(17), e13174. 10.1111/mcn.13174 PMC8189237 33719201

[fsn34201-bib-0008] Blaney, S. , Menasria, L. , Main, B. , Chhorvann, C. , Vong, L. , Chiasson, L. , Hun, V. , & Raminashvili, D. (2019). Determinants of undernutrition among young children living in Soth Nikum district, Siem Reap. Nutrients, 11(3), 685. 30909463 10.3390/nu11030685 PMC6471553

[fsn34201-bib-0009] Burns, J. , Catley, A. , Yusuf, M. M. , Timaado, F. , Lokono, P. , & Anete, J. (2022). Local knowledge and perceptions on the causes of malnutrition among the Dasanech in Kenya: A rapid participatory assessment in Illeret Ward, Marsabit County. USAID Nawiri Project. Nairobi https://fic.tufts.edu/assets/Nawiri_Illeret_Report2022‐7‐15Final.pdf

[fsn34201-bib-0010] Clarke, P. , Zuma, M. K. , Tambe, A. B. , Steenkamp, L. , & Mbhenyane, X. G. (2021). Caregivers' knowledge and food accessibility contributes to childhood malnutrition: A case study of Dora Nginza hospital, South Africa. International Journal of Environmental Research and Public Health, 18(20), 10691. 10.3390/ijerph182010691 34682438 PMC8535554

[fsn34201-bib-0011] de Onis, M. , Borghi, E. , Arimond, M. , Webb, P. , Croft, T. , Saha, K. , de‐Regil, L. M. , Thuita, F. , Heidkamp, R. , Krasevec, J. , Hayashi, C. , & Flores‐Ayala, R. (2019). Prevalence thresholds for wasting, overweight and stunting in children under 5 years. Public Health Nutrition, 22(1), 175–179. 10.1017/S1368980018002434 30296964 PMC6390397

[fsn34201-bib-0012] de Onis, M. , & Branca, F. (2016). Childhood stunting: A global perspective. Maternal & Child Nutrition, 12, 12–26. 10.1111/mcn.12231 27187907 PMC5084763

[fsn34201-bib-0013] Forshell, P. , & Wierup, M. (2006). Salmonella contamination: A significant challenge to the global marketing of animal food products. Revue Scientifique et Technique (International Office of Epizootics), 25(2), 541–554. 17094696

[fsn34201-bib-0014] Galgallo, S. O. (2017). Factors associated with nutritional status of children aged 6‐59 months in Maikona Ward of Marsabit County, Kenya. Master's thesis, University of Nairobi. http://erepository.uonbi.ac.ke/handle/11295/103352

[fsn34201-bib-0015] Geletaw, A. , Egata, G. , Weldgebreal, F. , Kibr, G. , & Semaw, M. (2021). Nutritional status and associated factors among primary schoolchildren from pastoral communities, Mieso‐Mulu District, Sitti zone, Somali Regional State, Eastern Ethiopia: Institution‐based cross‐sectional study. Journal of Nutrition and Metabolism, 2021, 6630620. 10.1155/2021/6630620 34603774 PMC8483933

[fsn34201-bib-0016] Iftikhar, A. , Bari, A. , Bano, I. , & Masood, Q. (2017). Impact of maternal education, employment and family size on nutritional status of children. Pakistan Journal of Medical Sciences, 33(6), 1401–1405. 10.12669/pjms.336.13689 29492067 PMC5768833

[fsn34201-bib-0017] Ireri, R. , Nyanchoka, A. , Mburu, M. , Ndungu, J. , & Kiarie, M. (2021). Determinants of nutrition status in children aged 6–59 months, in Kiandutu informal settlement, Thika, Kenya. Proceedings of the Nutrition Society, 80(OCE1), E3. 10.1017/S0029665121000045

[fsn34201-bib-0018] Kang, Y. , Aguayo, V. M. , Campbell, R. K. , Dzed, L. , Joshi, V. , Waid, J. L. , Gupta, S. D. , Haselow, N. J. , & West, K. P., Jr. (2018). Nutritional status and risk factors for stunting in preschool children in Bhutan. Maternal & Child Nutrition, 14(S4), e12653. 10.1111/mcn.12653 30412341 PMC6587444

[fsn34201-bib-0019] Kassa, T. , Meshesha, B. , Haji, Y. , & Ebrahim, J. (2016). Appropriate complementary feeding practices and associated factors among mothers of children age 6–23 months in Southern Ethiopia, 2015. BMC Pediatrics, 16, 131 (2016). 10.1186/s12887-016-0675-x 27542833 PMC4992197

[fsn34201-bib-0020] Khan, S. , Zaheer, S. , & Safdar, N. F. (2019). Determinants of stunting, underweight and wasting among children <5 years of age: Evidence from 2012–2013 Pakistan demographic and health survey. BMC Public Health, 19, 358. 10.1186/s12889-019-6688-2 30935382 PMC6444880

[fsn34201-bib-0021] Kinyoki, D. , Berkley, J. , Moloney, G. , Kandala, N. , & Noor, A. (2015). Predictors of the risk of malnutrition among children under the age of 5 years in Somalia. Public Health Nutrition, 18(17), 3125–3133. 10.1017/S1368980015001913 26091444 PMC4697134

[fsn34201-bib-0022] KNBS and ICF . (2023). Kenya demographic and health survey 2022. Key Indicators Report. KNBS and ICF.

[fsn34201-bib-0023] KNBS, MOH/Kenya . (2015). National AIDS Control Council/Kenya, KEMRI. Kenya Demographic and Health.

[fsn34201-bib-0024] Lukman, T. N. E. , Anwar, F. A. , Riyadi, H. , Harjomidjojo, H. , & Martianto, D. (2022). Responsive prediction model of stunting in toddlers in Indonesia. Current Research in Nutrition and Food Science Journal, 10(1), 302–310.

[fsn34201-bib-0025] Ma'alin, A. , Birhanu, B. , Melaku, S. , Tolossa, D. , Mohammed, Y. , & Kiros Gebremicheal, K. (2016). Magnitude and factors associated with malnutrition in children 6–59 months of age in Shinille woreda, Ethiopian Somali regional state: A cross‐sectional study. BMC Nutrition, 2, 44. 10.1186/s40795-016-0079-1

[fsn34201-bib-0026] Magnani, R. (1997). Sampling guide. FANTA. https://utsc.utoronto.ca/~kmacd

[fsn34201-bib-0027] Matonti, L. , Blasetti, A. , & Chiarelli, F. (2020). Nutrition and growth in children. Minerva Pediatrica, 72, 462–471. 32731734 10.23736/S0026-4946.20.05981-2

[fsn34201-bib-0028] Mbijiwe, J. , Ndung'u, Z. , & Kinyuru, J. (2022). Caregiver factors influencing nutritional status of preschool children in Mwingi west, Kitui County Kenya. Journal of Agriculture Science & Technology, 21(4), 22–34. 10.4314/jagst.v21i4.3

[fsn34201-bib-0029] Morakinyo, O. M. , Adebowale, A. S. , Obembe, T. A. , & Oloruntoba, E. O. (2020). Association between household environmental conditions and nutritional status of women of childbearing age in Nigeria. PLoS One, 15(12), e0243356. 33306726 10.1371/journal.pone.0243356 PMC7732090

[fsn34201-bib-0030] Mugenda, O. M. , & Mugenda, G. A. (1999). Research methods, quantitative and qualitative approach. Acts Press.

[fsn34201-bib-0031] Nankinga, O. , Kwagala, B. , & Walakira, E. J. (2019). Maternal employment and child nutritional status in Uganda. PLoS One, 14(12), e0226720. 10.1371/journal.pone.0226720 31856209 PMC6922416

[fsn34201-bib-0032] Nsubuga, E. J. , Arinda Kato, I. , Lee, S. , Ssenyondo, M. , & Isunju, J. B. (2022). Predictors of stunting and underweight among children aged 6 to 59 months in Bussi Islands, Wakiso District, Uganda: A cross‐sectional study. Nutrition and Metabolic Insights, 15, 11786388221125107. 36187343 10.1177/11786388221125107 PMC9520166

[fsn34201-bib-0033] Obeng‐Amoako, G. A. O. , Nangendo, J. , Karamagi, C. A. S. , Okiring, J. , Kiirya, Y. , Aryeetey, R. , et al. (2021). Factors associated with concurrent wasting and stunting among children 6–59 months in Karamoja, Uganda. Maternal & Child Nutrition, 17(1), e13074. 10.1111/mcn.13074 32830434 PMC7729532

[fsn34201-bib-0034] Okidi, L. , Duncan Ongeng, D. , Muliro, P. S. , & Matofari, J. W. (2022). Disparity in prevalence and predictors of undernutrition in children under five among agricultural, pastoral, and agro‐pastoral ecological zones of Karamoja sub‐region, Uganda: A cross sectional study. BMC Paediatrics, 22, 316. 10.1186/s12887-022-03363-6 PMC9150356 35637542

[fsn34201-bib-0035] Oniang'o, R. K. , Mutuku, J. M. , & Malaba, S. J. (2003). (2003). Contemporary African food habits and their nutritional and health implications. Asia Pacific Journal of Clinical Nutrition, 12(3), 331–336. 14505997

[fsn34201-bib-0036] Opiyo, R. O. (2018). Maternal, infant and young children nutrition knowledge attitude and practices baseline survey for Marsabit county, Nairobi.

[fsn34201-bib-0037] Paul, P. , & Saha, R. (2022). Is maternal autonomy associated with child nutritional status? Evidence from a cross‐sectional study in India. PLoS One, 17(5), e0268126. 10.1371/journal.pone.0268126 35544582 PMC9094570

[fsn34201-bib-0038] Poudel, S. , Adhikari, C. , Yadav, R. K. , Yadav, D. K. , Thapa, D. K. , & Jakovljevic, M. (2022). Disempowered mothers have undernourished children: How strong is the intrinsic agency? Frontiers in Public Health, 10, 817717. 35186848 10.3389/fpubh.2022.817717 PMC8850308

[fsn34201-bib-0040] Sinha, R. K. , Dua, R. , Bijalwan, V. , Rohatgi, S. , & Kumar, P. (2018). Determinants of stunting, wasting, and underweight in five high‐burden pockets of four Indian states. Indian Journal of Community Medicine, 43, 279–283. Survey 2014. Nairobi. http://statistics.knbs.or.ke 30662180 10.4103/ijcm.IJCM_151_18 PMC6319291

[fsn34201-bib-0041] Stamenkovic, Z. , Djikanovic, B. , Laaser, U. , & Bjegovic‐Mikanovic, V. (2016). The role of mother's education in the nutritional status of children in Serbia. Public Health Nutrition, 19(15), 2734–2742. 10.1017/S1368980016000768 27087502 PMC10270892

[fsn34201-bib-0042] Sulaiman, A. A. , Bushara, S. O. , Elmadhoun, W. M. , Noor, S. K. , Abdelkarim, M. , Aldeen, I. N. , Osman, M. M. , Almobarak, A. O. , Awadalla, H. , & Ahmed, M. H. (2018). (2018). Prevalence and determinants of undernutrition among children under 5‐year‐old in rural areas: A cross‐sectional survey in North Sudan. Journal of Family Medicine and Primary Care, 7, 104–110. 10.4103/jfmpc.jfmpc_73_17 PMC5958549 29915742

[fsn34201-bib-0043] UNICEF . (2019). The state of the World's children 2019. In Children, food and nutrition: Growing well in a changing world. UNICEF. https://www.unicef.org/reports/state‐of‐worlds‐children‐2019

[fsn34201-bib-0044] Weatherspoon, D. D. , Miller, S. , Ngabitsinze, J. C. , Weatherspoon, L. J. , & Oehmke, J. F. (2019). Stunting, food security, markets and food policy in Rwanda. BMC Public Health, 19, 882. 10.1186/s12889-019-7208-0 31272435 PMC6610945

[fsn34201-bib-0045] WHO . (2006). WHO child growth standards. World Health Organization. https://www.who.int/childgrowth/standards/Technical_report.pdf

[fsn34201-bib-0046] WHO, UNICEF, World Bank . (2021). Levels and trends in child malnutrition: key findings of the 2021 edition. https://www.who.int/publications/i/item/9789240025257

[fsn34201-bib-0047] WHO, UNICEF, World Bank . (2023). Levels and trends in child malnutrition: key findings of the 2021 edition. https://www.who.int/publications/i/item/9789240025257

[fsn34201-bib-0048] Win, H. , Shafique, S. , Mizan, S. , Wallenborn, J. , Probst‐Hensch, N. , & Fink, G. (2022). Association between mother's work status and child stunting in urban slums: A cross‐sectional assessment of 346 child‐mother dyads in Dhaka, Bangladesh (2020). Archives of Public Health, 80(1), 192. 10.1186/s13690-022-00948-6 35978414 PMC9382616

[fsn34201-bib-0049] Wolde, M. , Berhan, Y. , & Chala, A. (2015). Determinants of underweight, stunting and wasting among schoolchildren. BMC Public Health, 15, 1–9. 25595201 10.1186/s12889-014-1337-2 PMC4308904

[fsn34201-bib-0500] Yani, D. I. , Rahayuwati, L. , Sari, C. W. M. , Komariah, M. , & Fauziah, S. R. (2023). Family household characteristics and stunting: an update scoping review. Nutrients, 15(1), 233. 36615889 10.3390/nu15010233 PMC9824547

